# Improvement of long-term risks of cardiovascular events associated with community-based disease management in Chinese patients of the Xinjiang autonomous region of China

**DOI:** 10.1186/s12889-020-09157-8

**Published:** 2020-06-29

**Authors:** Yang Li, Cai Minzhang, Ma Minghui, Huang Xinmiao, Liu Laixin, Wang Bei, Zhu Weihai, Zhe Wei, Guan Yumei, Thitima Kongnakorn, Ying Xiao, Siyang Peng, David Hughes, Naranjargal Dashdorj, Thomas Hach

**Affiliations:** 1 School of Public Health, Peking University, No. 38 Xueyuan Road, Haidian District, 100191 Beijing China; 2 Health and Family Planning Commission of Xinjiang Uygur Autonomous Region, Urumqi, China, No.191 Longquan Street Tianshan Region, Urumqi, 830000 Xinjiang China; 3 Xinjiang Uygur Autonomous Region Center for Disease Control and Prevention, Urumqi, China, No.380, No.1 Jianquan Street Tianshan Region, Urumqi, 830000 Xinjiang China; 4 Novartis Pharmaceuticals China, No.4218 Jinke Road ZhangJiang Hi-Tech Park, Pudong, 201203 Shanghai China; 5 Shanxi Province Weinan City Center for Disease Control and Prevention, Weinan, China, No.5 Zhanbei Road, Weinan City, 714000 Shanxi China; 6 Evidera, Metro Building, 6th Floor, 1 Butterwick, London, W6 8DL UK; 7 Sandoz International AG, Lichtstrasse 35, 4056 Basel, Switzerland; 8 Onom Foundation, 3 Bogd Javzandamba 15 khoroo, Ulaanbaatar, 17011 Mongolia; 9 Novartis Pharma AG, Fabrikstrasse 12-1.03.14A, 4056 Basel, Switzerland

**Keywords:** Congestive heart failure (CHD), Cardiovascular disease (CVD), Community-based disease management (CBDM), Discrete-event simulation (DES) model, Hypertension, Stroke, Myocardial infarction (MI)

## Abstract

**Background:**

A recent community-based disease management (CBDM) pilot study reported a 20.5% prevalence of hypertension and a 0.5 and 3.6% prevalence of stroke and coronary heart disease (CHD), respectively, in an elderly population (mean age 65 years) in the Xin Jiang autonomous region of China. The CBDM was initiated in 2013 as an essential public health service; however, the potential long-term impact of CBDM on cardiovascular (CV: CHD and stroke) events is unknown. The objective of the study was to understand the long-term impact of CBDM interventions on CV risk factors using disease-model simulation based on a single-arm experimental study.

**Methods:**

A discrete event simulation was developed to evaluate the impact of CBDM on the long-term CV risk among patients with hypertension, in China’s Xin Jiang autonomous region. The model generated pairs of identical patients; one receives CBDM and one does not (control group). Their clinical courses were simulated based on time to CV events (CHD and strokes), which are estimated using published risk equations. The impact of CBDM was incorporated as improvement in systolic blood pressure (SBP) based on observations from the CBDM study. The simulation estimated the number of CV events over patients’ lifetimes.

**Results:**

During a 2-year follow up, the CBDM led to an average reduction of 8.73 mmHg in SBP from baseline, and a 42% reduction in smoking. The discrete event simulation showed that, in the control group, the model estimated incidence rates of 276, 1789, and 616 per 100,000 individuals for lifetime CHD, stroke, and CV-related death, respectively. The impact of CBDM on SBP translated into reductions of 8, 28, and 23% in CHD, stroke, and CV-related deaths, respectively. Taking into account CBDM’s reduction of both SBP and smoking, deaths from CHD, stroke, and CV-related deaths were reduced by 12, 30, and 26%, respectively.

**Conclusions:**

The implementation of CBDM in China’s Xinjiang autonomous region is expected to significantly reduce incidences of CHD, strokes, and CV-related deaths.

## Background

Cardiovascular diseases (CVD) are the leading causes of death for patients in both urban and rural areas of China. Currently, 44.6% of deaths in rural areas and 42.5% in urban areas are caused by CVDs. Hypertension is the primary risk factor for stroke and coronary heart disease (CHD). According to a national survey in China in 2012, the prevalence of hypertension in adults aged 18 years or older was 25.2%. It is estimated that 270 million Chinese patients are hypertensive and more than half of CVDs are associated with hypertension in China [1].

Awareness, treatment, and control rates of hypertension are still relatively low in China, at 26.1, 22.8, and 6.1%, respectively. Overestimated blood pressure (BP) control rates may contribute to therapeutic inertia and poor BP control among treated patients with hypertension [2, 3].

### Community based disease management

A community-based disease management (CBDM) pilot study was conducted to analyze the effect of follow-up management for chronic disease on community hypertension management in the Xinjiang Uygur Autonomous Region in China. The CBDM study reported a 20.5% prevalence of hypertension and 0.5 and 3.6% prevalence of stroke and CHD, respectively, in an elderly population (mean age 65 years). The CBDM was initiated in 2013 as an essential public health service; however, the potential long-term impact of CBDM on cardiovascular (CV) events is unknown.

The objective of the present study was to understand the long-term impact of CBDM interventions on CV risk factors and events using a simulation model, and to use disease-model simulation forecasts to predict how various interventions would affect CV outcomes such as stroke, CHD, and mortality. Risk factors considered included control of blood pressure, lipids, and smoking.

## Methods

### Community based disease management study

The CBDM study was initiated in 2013 in Xinjiang Uygur Autonomous Region. Of the 21.81 million local residents, 2800 patients with hypertension from 39 community and township hospitals were recruited with a stratified multistage random sampling method. Patients under the basic public health services management conforming to the following conditions were enrolled: (a) age ≥ 35 years, (b) suffering from primary hypertension, and (c) participating in this management program of hypertensive patients, which acts as a basic public health service. This was a single-arm experimental study; all patients received interventions for BP, which included medication, life-style management, dietary recommendations, and exercise. Risk factors that could influence the outcome of hypertension control were captured at baseline and at the end of the two-year follow-up, including but not limited to, age, gender, BP, smoking status, diabetes status, and history of CVD. The CBDM population and the efficacy of the intervention such as systolic BP reduction and smoking reduction were used to inform the simulation model.

The study was approved by the ethics committees of the Peking University, School of Public Health, and the study was conducted in accordance with the International Council on Harmonization (ICH, E6) guidelines and Declaration of Helsinki and its subsequent revisions. The data was collected by the Xinjiang CDC and the written patient consents were received prior to the survey.

### Discrete-event simulation model

A discrete-event simulation (DES) model was developed in Microsoft Excel^®^ to evaluate the impact of CBDM on long-term CV (CHD and stroke) risks among patients with hypertension. The DES approach for disease modeling is a well-established technique that is in agreement with the modeling good research practices recommended by the International Society for Pharmacoeconomics and Outcomes Research (ISPOR), and is widely used in published models [4–6]. The model was designed to simulate clinical events associated with high BP, including acute myocardial infarction (MI), coronary death, stroke, stroke-related death, and background mortality over a 30-year time horizon. DES was selected because of the dynamic nature of events and unpredictable timing of disease- and treatment-related events. This approach used time-to-event calculations to simulate the clinical course of these patients. The core of this model was a set of equations representing the physiological pathways pertinent to diseases and their complications. Depending on the baseline characteristics, each patient followed a distinct disease pathway. The event rate and life-year outcome for the overall modeled cohort and longitudinal event outcomes were reported.

At the beginning of each simulation, individual patients were created by sampling from patient profiles of those who participated in the CBDM study. Each patient profile included age, gender, systolic blood pressure (SBP), total cholesterol, high-density lipoprotein (HDL), diabetes status, smoking status, and body mass index (BMI). In addition, the disease history of stroke, CVD, and CHD were also assigned to each patient at baseline. After the patients were created, they were “cloned” to produce two identical cohorts. This ensured that the comparisons were not affected by differences in baseline characteristics. One patient in each pair was assumed to experience changes in CV (CHD and stroke) risk factors due to CBDM intervention (including changes in SBP, lipid levels, and smoking cessation), while the other patient was assumed to receive no intervention. The impact of intervention was assumed to take place within the first 3 months after entering the model and to last for the entire model time horizon. Any exacerbation of the baseline condition was not considered. Patients receiving no intervention were assumed to have no change in these risk factors, except for age, throughout the model time horizon. Age and gender-specific background mortality was considered to account for the health deterioration of a general population.

Each patient was exposed to various risks: CHD (acute MI and coronary death), stroke, and background death, which were handled as competing risks. The competing risks were implemented by using a series of risk equations to translate the patient’s risk factors (e.g. age, gender, and SBP) into a distribution of failure times that were specific to the individual. A time for each event can then be assigned to each individual by sampling from the corresponding distribution. The prediction equations for CHD (i.e. acute MI and coronary death) were obtained from the Chinese Multi-Provincial Cohort Study (CMCS), with risk factors including age, BP, total cholesterol, HDL, diabetes, and smoking (Table 1) [7]. The CMCS was conducted in 30,121 Chinese adults aged from 35 to 64 with a follow-up of 10 years (1992 to 2002) for CHD events. The CMCS cohort had 191 hard CHD events (including coronary death and MI) and 625 total deaths.

**Table 1:** Variables of CHD risk equation used in model base case

	Male	Female
	Coefficient	Coefficient
**Age**	0.07	0.07
**SBP - optimal**	−0.51	−0.5
**SBP – normal**	Reference	Reference
**SBP – high normal**	0.21	−0.87
**SBP – stage 1 hypertension**	0.33	0.34
**SBP – stage 2–4 hypertension**	0.77	0.47
**Total cholesterol < 160 mg/dL**	−0.51	0.18
**Total cholesterol 160–199 mg/dL**	Reference	Reference
**Total cholesterol 200–239 mg/dL**	0.07	0.13
**Total cholesterol 240–279 mg/dL**	0.32	0.14
**Total cholesterol ≥ 280 mg/dL**	0.52	1.67
**HDL-C < 35 mg/dL**	−0.25	0.62
**HDL-C 35–44 mg/dL**	0.01	0.3
**HDL-C 45–49 mg/dL**	Reference	0.08
**HDL-C 50–59 mg/dL**	−0.07	Reference
**HDL-C ≥ 60 mg/dL**	−0.4	−0.78
**Diabetes (yes = 1, no = 0)**	0.09	0.18
**Smoking (yes = 1, no = 0)**	0.62	− 0.95
**Survival**	0.9895	0.9961

*CHD* coronary heart disease, *HDL-C* high-density lipoprotein cholesterol, *SBP* systolic blood pressure

SBP optimal: ≤120 mmHg; SBP normal 120–130 mmHg; SBP high normal 130–140 mmHg; SBP stage 1 hypertension 140–160 mmHg; SBP stage 2–4 hypertension ≥160 mmHg

The prediction equation for stroke was obtained from the People’s Republic of China - United States of America (PRC-USA) study with risk factors including age, BP, BMI, total cholesterol, smoking, and diabetes (Table 2) [8]. PRC-USA collaborative study was conducted in 9903 participants with 17 years of follow-up until the year of 2000; 266 strokes and 105 CHD events occurred.

**Table 2:** Variables of stroke risk equation used in model base case

	Male	Female
	Coefficient	Coefficient
**Age**	0.07	0.09
**SBP < 120**	−0.55	−0.83
**SBP 120–129**	Reference	Reference
**SBP 130–139**	0.40	0.23
**SBP 140–159**	0.81	0.80
**SBP 160–179**	1.70	1.37
**SBP ≥ 180**	2.53	1.85
**BMI < 24**	Reference	Reference
**BMI ≥ 24**	0.29	0.68
**Total cholesterol < 3.62 mmol/L**	Reference	Reference
**Total cholesterol 3.62–5.16 mmol/L**	−0.01	−0.09
**Total cholesterol ≥ 5.17 mmol/L**	0.30	0.27
**Smoking**	0.71	0.47
**Diabetes**	0.07	0.96
**Survival**	0.9835	0.9948
**Survival**	0.9895	0.9961

*BMI* body mass index, *SBP* systolic blood pressure

Twenty-eight day case fatality rates for CHD and stroke were estimated based on the Sino-MONICA study for the Chinese population (Table 3) [9]. Background mortality was obtained from the life table for China [10, 11]. The time-to-event calculations were estimated at baseline. The event that was estimated to occur first was recorded by the model and triggered the update of patient characteristics and estimation of the time-to-next event. Other than the time that the events occurred, all time-to-event and clinical measurements were updated in the model based on a quarterly time unit for the first 2 years, then based on an annual time unit beyond 2 years until the end of model time horizon. The quarterly time unit was intended to provide granularity that was consistent with the quarterly assessment schedule during the two-year follow-up of the CBDM study.

**Table 3:** Case fatality rate for CHD and stroke, by gender and age group

	CHD		Stroke	
	Male	Female	Male	Female
**35–44 years, %**	30	50	6	10
**45–54 years, %**	38	42	11	10
**55–64 years, %**	41	43	17	15
**65–74 years, %**	45	51	27	25
**75–84 years, %**	53	57	31	28

*CHD* coronary heart disease

### Study outcomes and analysis

A cohort of virtual patients that represented the patient population of 2800 from the CBDM study was generated. Each simulated patient was run through the baseline control and intervention scenarios for 30 iterations over a 30-year time horizon.

Three intervention scenarios were explored in the model, including 1) SBP reduction only, 2) SBP reduction and smoking cessation, and 3) SBP reduction, smoking cessation, and lipid control. Clinical inputs of SBP reduction and smoking cessation were obtained from the clinical results of the CBDM study. Lab values for lipid status were not available from the CBDM study. Therefore, based on CBDM physician suggestion, a 10% increase in HDL and a 10% decrease in total cholesterol from the baseline was assumed for lipid control. The following health outcomes generated from the DES model were (1) total event count and event rate of CHD, (2) total event count and event rate of stroke, (3) mortality due to CHD, (4) mortality due to stroke and (5) total death.

Subgroup analysis was also conducted for patients aged < 60 years versus patients aged ≥60 years, male versus female, and patients with SBP < 140 mmHg versus patients with SBP ≥140 mmHg.

## Results

### Clinical outcomes of CBDM study

Baseline population characteristics of the Chinese population from the CBDM study that were simulated in the DES model are reported in Table 4. The characteristics were well balanced between male and female, except for smoking status. Smoking was more prevalent in males (22%) than in females (1%).

**Table 4:** Baseline population characteristics

	Full population (***N*** = 2800)	Male (***N*** = 1199)	Female (***N*** = 1601)
**Age**	65.4 (11.1)	65.4 (11.4)	65.4 (10.8)
**SBP (mmHg), mean (SD)**	144.3 (20.0)	143.9 (20.1)	144.6 (20.0)
**SBP < 120 mmHg, %**	4	4	4
**SBP 120–129 mmHg, %**	13	13	13
**SBP 130–139 mmHg, %**	19	20	19
**SBP 140–159 mmHg, %**	40	40	40
**SBP 160–179 mmHg, %**	17	18	16
**SBP ≥ 180 mmHg, %**	7	7	7
**Total cholesterol (mg/dL), mean (SD)**	212.3 (22.0)	213.3 (21.5)	211.5 (22.3)
**HDL (mg/dL), mean (SD)**	67.4 (6.2)	69.6 (11.1)	65.8 (5.3)
**LDL (mg/dL), mean (SD)**	117.9 (16.5)	117.8 (16.2)	118.1 (16.6)
**Female, %**	57	0	100
**Diabetes, %**	16	14	19
**Smoking, %**	11	22	1

*HDL* high-density lipoprotein, *LDL* low-density lipoprotein, *SBP* systolic blood pressure, *SD* standard deviation

For the overall population, the intervention program in the CBDM study was found to be associated with a reduction of 8.73 mmHg in SBP and 42% reduction of smoking. Table 5 presents the SBP reduction by subgroups from the CBDM study. SBP reduction over the two-year follow-up was higher in females (9.28 mmHg) than in males (7.94 mmHg). SBP reduction between the < 60- and ≥ 60-year age groups were similar. The subgroup with baseline SBP ≥140 mmHg was reported to have the highest SBP reduction of 18.28 mmHg.

**Table 5:** SBP reduction in subgroups

Subgroups	Sample size	SBP reduction (mmHg)	
		Mean	SD
**Male**	1199	−7.9436	25.3237
**Female**	1601	−9.2856	25.2491
**Age < 60**	890	−8.9761	26.3045
**Age ≥ 60**	1910	−8.4864	24.5032
**SBP < 140 mmHg**	1014	8.9768	15.7911
**SBP ≥ 140 mmHg**	1786	−18.2826	16.7164

*SBP* systolic blood pressure, *SD* standard deviation

### Long-term projection of clinical outcomes in overall population

The CBDM population with hypertension intervention was found to be associated with significant improvement in simulated 30-year risk across all clinical outcomes. In the overall population, the impact of CBDM on SBP resulted in reductions of 8, 28, 5, and 28% in CHD events, strokes, CHD-related deaths, and stroke-related deaths, respectively. Taking into account CBDM’s reduction of both SBP and smoking, CHD, stroke, CHD-related deaths and stroke-related deaths fell by 12, 30, 9, and 30%, respectively. Event rates were further reduced when smoking cessation and lipid control in terms of increased HDL and decreased total cholesterol, were applied (20, 46, 18, and 45% for CHD, stroke, CHD-related deaths and stroke-related deaths, respectively). The event rate per 100,000 people simulated with the overall population over the 30-year time horizon is presented in Table 6**.**

**Table 6:** Event rate per 100,000 people in overall population

Intervention	CHD	Stroke	CHD-related death	Stroke-related death
**Without intervention**	276.0	1788.9	139.4	476.2
**SBP**	253.8	1291.0	131.6	342.8
**SBP + smoking cessation**	242.1	1255.0	125.7	332.7
**SBP + smoking cessation + lipid control**	218.1	960.0	112.7	259.3

*CHD* coronary heart disease, *SBP* systolic blood pressure

**Fig. 1:**
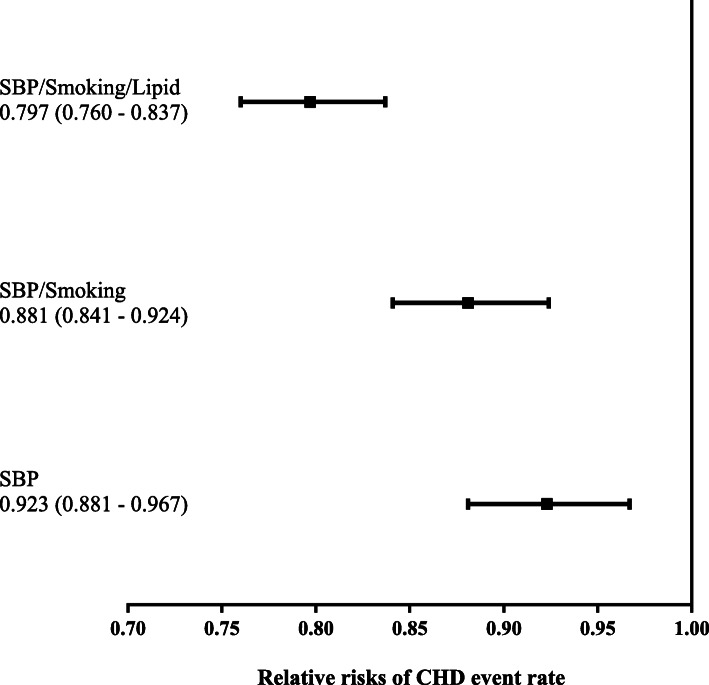
Relative Risk of CHD Event. SBP/smoking/lipid indicates that the combined effect of all three interventions was able to reduce all three risk factors. SBP/smoking indicates that the combined effect of both interventions was able to reduce both risk factors. *CHD* coronary heart disease, *SBP* systolic blood pressure

**Fig. 2:**
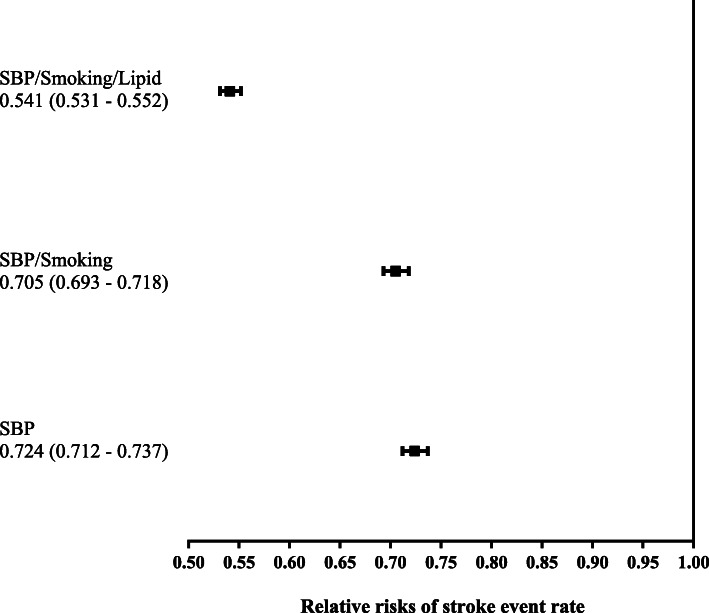
Relative Risk of Stroke Event. SBP/smoking/lipid indicates that the combined effect of all three interventions was able to reduce all three risk factors.SBP/smoking indicates that the combined effect of both interventions was able to reduce both risk factors. *CHD* coronary heart disease, *SBP* systolic blood pressure

Figures 1 and 2 present the relative risks of clinical events between the three scenarios with interventions versus the one scenario without intervention. The relative risks and their 95% CI of intervention group versus no intervention group for CHD and for stroke were lower than one for all intervention scenarios indicating significant risk reduction with the intervention group. Risk reduction was greatest (20 and 46% reduction in CHD and stroke, respectively) when impact of intervention on SBP, smoking cessation, and lipid profiles were considered.

**Fig. 3:**
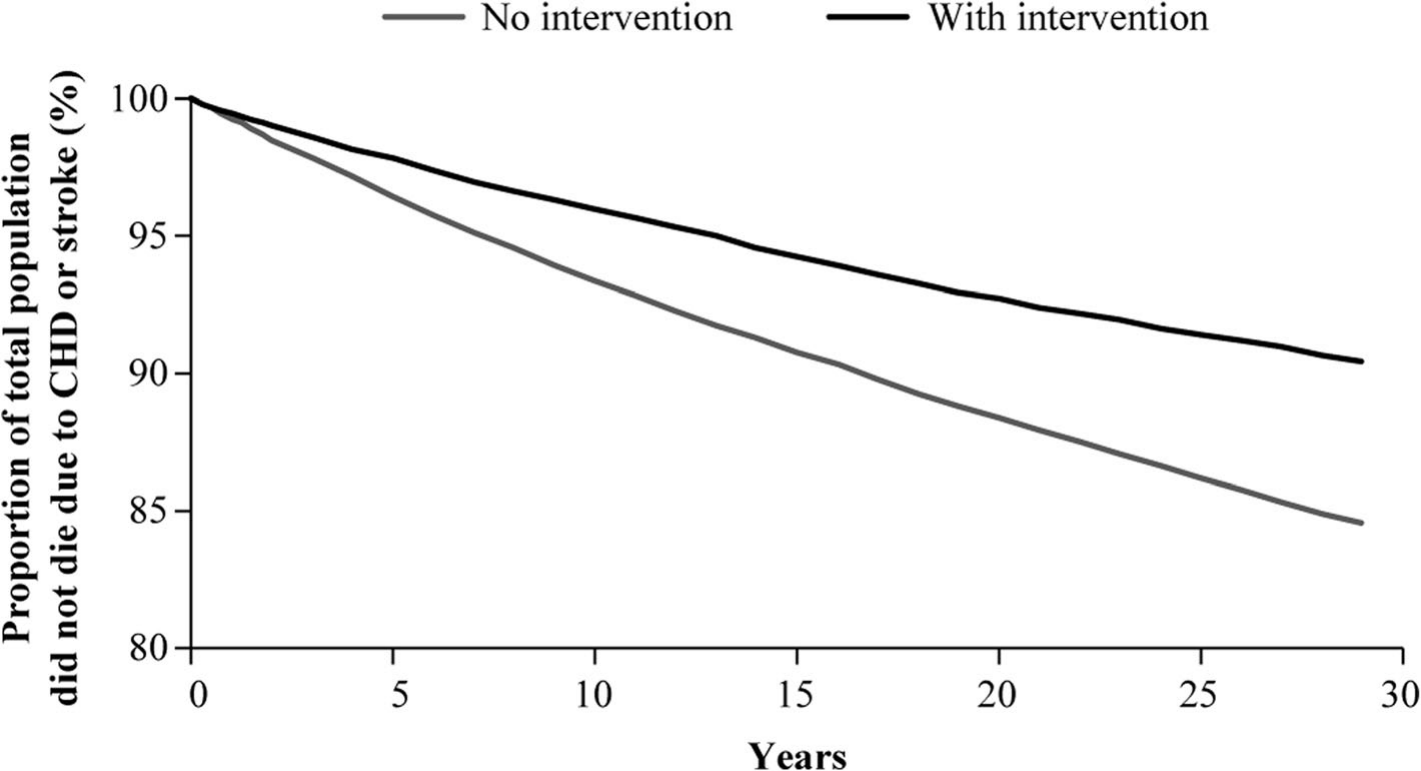
Impact of SBP reduction, smoking cessation, and lipid control on mortality due to CHD or stroke. *CHD* coronary heart disease, *SBP* systolic blood pressure

Figure 3 illustrates the impact of intervention in the CBDM study on mortality due to CHD or stroke over a 30-year time horizon. Compared to the scenario without intervention, the addition of SBP control, smoking cessation, and lipid control contributed to a reduction of mortality due to CHD or stroke.

### Long-term projection of clinical outcomes in subgroups

Subgroup analysis of long-term projection of clinical outcomes was conducted in the CBDM population aged < 60 years versus ≥60 years, male versus female, and baseline SBP < 140 mmHg versus baseline SBP ≥140 mmHg.

The subgroup analysis results showed that the CBDM population with hypertension intervention was associated with improvement in simulated 30-year risk across all clinical outcomes (CHD, stroke, and death related to CHD and stroke) in all subgroups with an exception in the subgroup with baseline SBP below 140 mmHg across all clinical outcomes. The greatest reduction in event rates was seen in the subgroup with SBP ≥140 mmHg. Patients aged < 60 years, male, or SBP ≥140 mmHg were reported to benefit more from the hypertension control program over the long term than older patients, females, or low SBP subgroups, respectively. Table 7 presents the event rates per 100,000 people and relative risks of CHD, stroke, CHD-related death, and stroke-related death across all subgroups.

**Table 7:** Event rate per 100,000 people and relative risk in subgroups

Subgroup	Intervention	CHD		Stroke		CHD-related death^**a**^		Stroke-related death^**a**^	
		Event rate	Relative risk	Event rate	Relative risk	Event rate	Relative risk	Event rate	Relative risk
**Age ≥ 60 years**	**Without intervention**	404.1		2568.5		210.2		720.2	
	**SBP**	370.2	0.92	1892.0	0.74	195.7	0.94	536.3	0.75
**Age < 60 years**	**Without intervention**	150.3		918.9		70.2		209.6	
	**SBP**	125.6	0.84	632.8	0.69	60.0	0.85	146.4	0.70
**Gender, female**	**Without intervention**	149.9		1875.9		81.0		491.7	
	**SBP**	143.9	0.96	1378.8	0.74	76.6	0.95	361.5	0.74
**Gender, male**	**Without intervention**	455.6		1679.3		218.2		471.9	
	**SBP**	410.0	0.91	1172.1	0.70	193.6	0.89	332.4	0.71
**SBP ≥ 140mmHg**	**Without intervention**	340.0		2393.8		173.0		637.6	
	**SBP**	270.2	0.80	1331.4	0.56	137.6	0.81	351.9	0.56
**SBP < 140mmHg**	**Without intervention**	174.6		800.2		85.7		216.8	
	**SBP**	223.9	1.28	910.8	1.14	110.8	1.29	245.6	1.13

*CHD* coronary heart disease, *SBP* systolic blood pressure

^a^Acute death determined by 28-day case fatality rates

## Discussion

This study aimed to evaluate the potential impact of improved management of hypertension and other known CV (CHD and stroke) risk factors (dyslipidemia and smoking) in China over a 30-year time horizon. Our analysis showed that BP intervention implemented in the CBDM study, which included medication, life-style management, dietary recommendations, and exercise can lead to a 20% risk reduction in CHD events and 45% risk reduction in stroke events. The addition of smoking cessation and dyslipidemia control would further reduce the number of CHD and stroke events. As stroke is generally considered more dangerous than a CHD event this may have led to underreporting of stroke prevalence in the pilot study. Hence, it seems even more important to foster education and preventive measures.

Along with the clarion call for expanding Universal Health Coverage globally [12], the World Health Organization (WHO) has also emphasized the urgency of strengthening primary care services worldwide [13]. In addition, the management of hypertension, one of the most common chronic health conditions and the number one risk factor for overall mortality, has been suggested as a proxy measure of clinical performance [14, 15].

Dyslipidemia and smoking control were assessed as additional interventions to hypertension control. The further reduction in CV (CHD and stroke) events highlights the importance of a multifactorial approach to target CV (CHD and stroke) event reduction. The results give a clear indication of the potential gains in mortality and morbidity by addressing three of the common and treatable risk factors.

The CBDM population represented an older and more hypertensive population in the Xinjiang province. An epidemiological survey that enrolled 14,618 adults aged ≥35 years from Xinjiang found that 42% of the population had SBP > 140 mmHg [16], whereas in the CBDM population, over 60% of patients had SBP > 140 mmHg. In comparison with the general population in China, the CBDM population had higher total cholesterol (212 mg/dL vs 174 mg/dL) and higher HDL (67 mg/dL vs 46 mg/dL) [17], smoking was less prevalent in the CBDM population than in the general Chinese population (52.1% in male and 2.7% in female) [18]. If CBDM could be applied to the total population of Xinjiang Uygur Autonomous Region (21.8 million), this approach could save approximately 27,000 deaths from CHD or stroke each year. In China, there were 2.04 million deaths due to hypertension in 2010 [19]. The casualty is increasing over time. Applying the intervention of our CBDM study even more broadly in China could save many lives.

Given the small proportion of smokers (11% reported from CBDM study) in the baseline population and the smoking cessation rate of 42% through the intervention program, the smoking population was too small in the study to capture significant impact on incidence of stroke.

Based on our CBDM experience, we suggest investigating further, how the effects of hypertension management differ between urban and rural areas, between different insurance schemes, or different management schemes (i.e. health management only, health management plus basic medical services model or integrated care model).

This analysis has several strengths. The level of BP control in the model simulation was based on the observation in the real-world CBDM study. In addition, based on a DES model, the analysis captured change in CV risk factors at the individual level and more accurately estimated the impact on CV event risk. The DES model also accounted for the elevation of future event risks when a CV (CHD or stroke) event occurs.

The impact of hypertension control on long-term clinical outcomes in our study is consistent with the previous findings. The similar results were proven in a Chinese prospective cohort study with 5006 eligible hypertensive patients aged 60 years or older during 4.8 years of follow-up [20]. Compared with the reference group of BP < 140/90 mmHg, the risks of all-cause death, CV death, and stroke were significantly increased in the group with BP of 140–149/< 90 mmHg [20]. A similar trend was also reported in the study by Stevens et al. [21].

We recognize that there are limitations to this analysis. First, the analysis was based on risk equations that were developed from older data and a different population. It is possible that the equations were not able to capture the impact of some risk factors in certain subpopulations and led to uncertainty in some of the subgroup results. In addition, the efficacy of intervention was based on 2 years of data. The effect of intervention was assumed to remain stable for the entire model time horizon. In reality, there may be a lack of adherence to the management of CV (CHD and stroke) risk factors not captured in the model, and treatment effect may fluctuate. Last, and most importantly, deaths due to CHD or stroke were captured only by the 28-day case fatality rates. Long-term impact of CHD and stroke on mortality was not modeled due to data limitations. Had the model captured long-term impact of CHD and stroke on mortality, the clinical benefits of the intervention are expected to be magnified. Despite the limitations, the analysis was able to provide a range of estimation that will shed light on the impact of hypertension control in the real-world practice.

## Conclusion

The implementation of the CBDM study in China’s Xinjiang Uygur Autonomous Region is expected to significantly reduce the incidence of CV events, strokes, and CV-related deaths.

## Data Availability

The datasets used and/or analyzed during the current study are available from the corresponding author on reasonable request.

## Abbreviations

BMI: Body mass index
BP: Blood pressure
CBDM: Community-based disease management
CHD: Coronary heart disease
CMCS: Chinese Multi-Provincial Cohort Study
CV: Cardiovascular
CVD: Cardiovascular diseases
DES: Discrete-event simulation
HDL-C: High-density lipoprotein cholesterol
ISPOR: International Society for Pharmacoeconomics and Outcomes Research
MI: Myocardial infarction
PRC-USA: People’s Republic of China - United States of America
SBP: Systolic blood pressure
WHO: World Health Organization
